# Correlation of Vitreous Vascular Endothelial Growth Factor and Uric Acid Concentration Using Optical Coherence Tomography in Diabetic Macular Edema

**DOI:** 10.1155/2015/478509

**Published:** 2015-11-17

**Authors:** Libuse Krizova, Marta Kalousova, Ales Antonin Kubena, Oldrich Chrapek, Barbora Chrapkova, Martin Sin, Tomas Zima

**Affiliations:** ^1^Institute of Medical Biochemistry and Laboratory Diagnostics, First Faculty of Medicine, Charles University in Prague and General University Hospital, U Nemocnice 2, 128 08 Prague 2, Czech Republic; ^2^Augenzentrum Augsburg, Prinzregentenstraße 25, 86150 Augsburg, Germany; ^3^Department of Ophthalmology, Faculty of Medicine and Dentistry, Palacky University Olomouc, I. P. Pavlova 6, 775 20 Olomouc, Czech Republic

## Abstract

*Purpose*. We investigated two factors linked to diabetic macular edema (DME), vitreous and serum levels of vascular endothelial growth factor (VEGF) and uric acid (UA) in patients with DME, and compared the results with changes in optical coherence tomography (OCT) and visual acuity (VA).* Methods*. A prospective study of 29 eyes, 16 cystoid DME and nonproliferative diabetic retinopathy (DR) and 13 nondiabetic controls. Biochemical analysis of vitreous and serum samples was performed and OCT scans were graded according to central retinal thickness (CRT), cube volume (CV), cube average thickness (CAT), and serous retinal detachment (SRD).* Results*. In DME group, intravitreal concentrations of VEGF (*p* < 0.001), UA (*p* = 0.038), and total protein (*p* < 0.001) were significantly higher than in control group. In DME subjects, intravitreal UA correlated significantly with intravitreal VEGF (*ƍ* = 0.559, *p* = 0.03) but not with total vitreous protein and serum UA. Increased intravitreal VEGF in DME group correlated with increase in CV (*ƍ* = 0.515/*p* = 0.041). None of the OCT parameters correlated with the VA.* Conclusions*. The results suggest that the CV might be assessor of anti-VEGF therapy efficacy. Second, apart from VEGF, the role of UA in the pathogenesis and progression of DR should be considered.

## 1. Introduction

Diabetic macular edema (DME) is a common complication of diabetic retinopathy (DR) and a leading cause of visual loss in this population [1, 2]. Major components of DME are retinal microvascular dysfunction and blood-retinal barrier (BRB) breakdown with consequent increase in vascular permeability that allows plasma compounds to leak into the retina [3–5]. There is evidence that upregulation of angiogenic and inflammatory factors, including vascular endothelial growth factor (VEGF), and downregulation of antiangiogenic factors as well as redox shift contribute to the breakdown of the BRB in DR [5–10]. Oxidative stress and inflammation also play an important role in the pathogenesis of DR and DME [5].

VEGF causes conformational changes in the tight junctions of the retinal vascular endothelial cells and plays a major role in the increased vascular permeability and BRB breakdown in diabetic eyes [5, 7, 11, 12]. Vitreous VEGF levels correlate significantly with the severity of DR [13], but DME can occur in nonproliferative DR (NPDR) as well as proliferative DR (PDR). Interventional studies on ranibizumab, a monoclonal antibody against VEGF, have shown that intraocular injections of ranibizumab significantly reduce foveal thickness and improve visual acuity in patients with DME [14, 15]. This demonstrates that VEGF is an important therapeutic target in DME. However, the conclusions of the studies were not based on the comparison of the real intravitreal concentration of VEGF with the foveal thickness in OCT; they only asses the retinal thickness before and after therapy.

In our earlier study, we found that also the intravitreal uric acid (UA) concentrations correlated significantly with degree of DR [16]. We suspect that UA may play a role in the pathogenesis of DR and DME: studies of UA strongly suggest that its redox potential affects endothelial function [17] and might contribute to the BRB breakdown. The correlation of intravitreal UA with VEGF in NPDR and DME has not been studied yet.

Optical coherence tomography (OCT) has enabled clinicians to noninvasively evaluate the effect of DR on retinal thickness in a standard clinical setting [18, 19]. However, there are very limited data on how OCT parameters in DME correlate with vitreous levels of VEGF and other biochemical parameters.

The aim of our study was to analyse the vitreous and serum of diabetic patients with DME and severe NPDR and compare them to nondiabetic controls. The analysis focused on VEGF and UA as two possible pathogenetic factors in the development of DME. We compared blood and vitreous levels of VEGF, UA, and protein between the two study groups and describe their correlation with the changes seen in OCT.

## 2. Materials and Methods

### 2.1. Subjects

This consecutive, prospective study involved 29 patients divided into two groups. First group involved 16 subjects with type 2 diabetes mellitus (DM) with NPDR and cystoid DME. In this group, the mean duration of DM was 18.6 ± 8.3 years and 15 patients (93.75%) were treated with insulin and 1 patient (6.25%) was treated with peroral antidiabetics. A group of 13 nondiabetic subjects with idiopathic epiretinal membrane and diffuse retinal thickening served as control. Characteristics of all subjects are listed in Table 1.

The diagnosis of DM was based on the WHO criteria [20]. DM duration was defined as the duration from the first diagnosis of DM to the time of vitreous sampling. All patients underwent a standard ophthalmologic examination including measurement of best corrected visual acuity, slit-lamp biomicroscopy, indirect ophthalmoscopy, and OCT. The retinopathy was graded according to the Early Treatment Diabetic Retinopathy Study Research Group and patients enrolled in the study had moderate to severe nonproliferative DR (NPDR) [21]. The center involving DME was defined clinically and confirmed by retinal thickening in cross-sectional spectral domain (SD) OCT scans. The indications for vitrectomy in this study were macular edema and preoperative best corrected visual acuity (BCVA) more than 0.3 logMAR (logarithm of the minimum angle of resolution) and in the diabetic group no or poor response to previous therapy with photocoagulation or intravitreal injection. Exclusion criteria were as follows: (a) history of intraocular haemorrhage, (b) prior vitreoretinal surgery, (c) other ocular surgeries or laser coagulation less than 6 months prior to the operation, (d) history of ocular inflammation, (e) proliferative DR or other retinal conditions causing neovascularisation, (f) ophthalmic disorders associated with macular edema, and (g) treatment with intravitreal anti-VEGF or steroid injections (e.g., triamcinolone, dexamethasone, bevacizumab, ranibizumab, and aflibercept) less than 6 months prior to the operation.

At the time of the study, all patients were in a stable clinical condition without clinical or laboratory signs of acute inflammation. The research was approved by the Local Institutional Ethics Committee, Faculty of Medicine and Dentistry, Palacky University Olomouc, Czech Republic. Data and sample collection was independent of all treatment decisions. It did not affect a patient's access to treatment and fully complied with all ethical and legal requirements for noninterventional data collection in the Czech Republic. All patients gave written informed consent to the treatment, as well as data collection. The reported investigations were in accordance with the principles of the current version of the Declaration of Helsinki.

### 2.2. Methods

OCT examinations were performed one day before vitrectomy with spectral domain OCT (Cirrus HD-OCT, Carl Zeiss Meditec AG, Jena, Germany) using macular cube acquisition according to the manufacturer's protocol. The macular cube 512 × 128 scan consists of 128 raster scans with 512 A-scans, within a 6 × 6 mm macular area. The mean central retinal thickness (CRT, i.e., central subfield thickness) from the internal limiting membrane to the retinal pigment epithelium at the fovea was defined as the mean retinal thickness in a 1 mm diameter circular zone concentred on the fovea. Also cube volume (CV) and cube average thickness (CAT) of the scanned area were calculated by Cirrus HD-OCT software and checked for accuracy. The CV is calculated from the 1 mm diameter zone and CAT from the central 6 mm diameter zone concentred on the fovea.

Based on previous studies that evaluated morphological changes in DME [22, 23], the central scan through the fovea was assessed for the presence of intraretinal cysts and serous retinal detachment (SRD) by an independent examiner.

Vitrectomy was performed to improve visual acuity and to decrease retinal thickness in the macula. Each patient underwent standard three-port therapeutic pars plana vitrectomy using current surgical techniques (the Alcon CONSTELLATION Vision System). Before opening the infusion port at the start of the vitrectomy, undiluted vitreous samples were obtained and collected in sterile tubes (cca. 0.3 mL). Overnight fasting blood samples were drawn from the antecubital vein at the time of vitrectomy and used for biochemical assay. Samples of vitreous and serum were rapidly frozen after collection at −80°C.

Routine biochemical parameters of serum were determined by standard clinical-chemistry methods. The concentration of UA was estimated using enzymatic methods (uricase-peroxidase) with photometric detection (Modular, Roche, Germany). The low detection limit of the method was 30 *μ*mol/L. HbA_1c_ was measured by high performance liquid chromatography and calibration was traced to the reference method of the International Federation of Clinical Chemistry (Variant II, Bio-Rad; http://www.bio-rad.com/). The concentration of VEGF was quantified by enzyme linked immunosorbent assay (ELISA) using a commercial human VEGF Kit (R and D Systems, Minneapolis, MN, USA) according to the manufacturer's protocol. The limits of Quantification for VEGF were min = 31.2 pg/mL and max = 1000 pg/mL, respectively.

### 2.3. Statistical Analysis

All statistical analyses were performed using the SPSS version 16 (SPSS Inc., Chicago, IL, USA). We calculated the median with 1st and 3rd quartile (IQR, interquartile range). In 16 subjects, the intravitreal VEGF and in 3 subjects the intravitreal UA concentration were under the detection limit; these subjects were included in the statistical analysis to avoid selection bias. Hence, we used the nonparametric analysis for ordinal variables, and the concentrations under the detection limit were assigned “minor than other.” The comparison between DME group and control group was done by Mann-Whitney *U* test and Fisher's exact test. To examine correlations, Spearman rank correlation coefficients were calculated. Two-tailed *p* values of less than 0.05 were considered significant.

## 3. Results and Discussion

### 3.1. Results

#### 3.1.1. Biochemical Analysis of Serum and Vitreous

**Figure 1:**
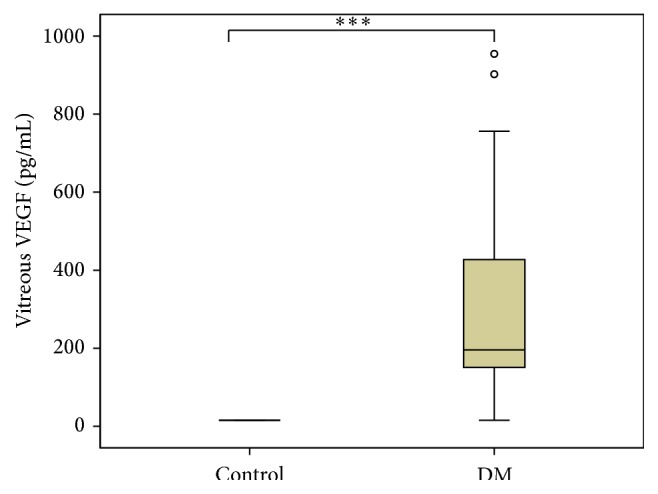
Vitreous concentrations of VEGF in diabetic versus control group. DM group *n* = 16, control group *n* = 13, and ^*∗∗∗*^ *p* < 0.001 DM versus control patients.

**Figure 2 fig2:**
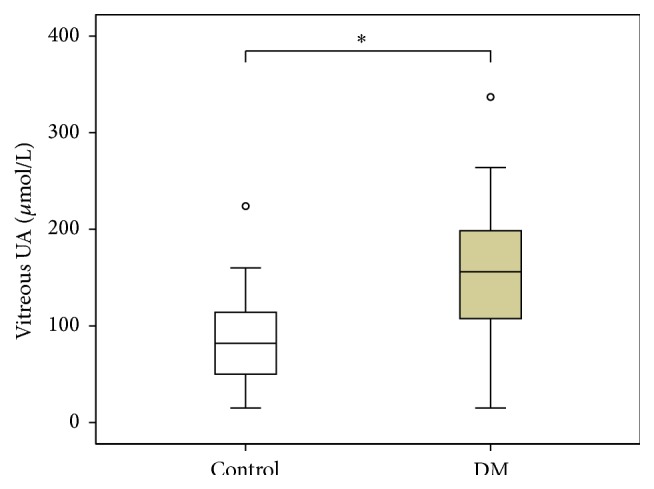
Vitreous concentrations of uric acid in diabetic versus control group. DM group *n* = 16, control group *n* = 13, and ^*∗*^ *p* = 0.038 DM versus control patients.

**Figure 3:**
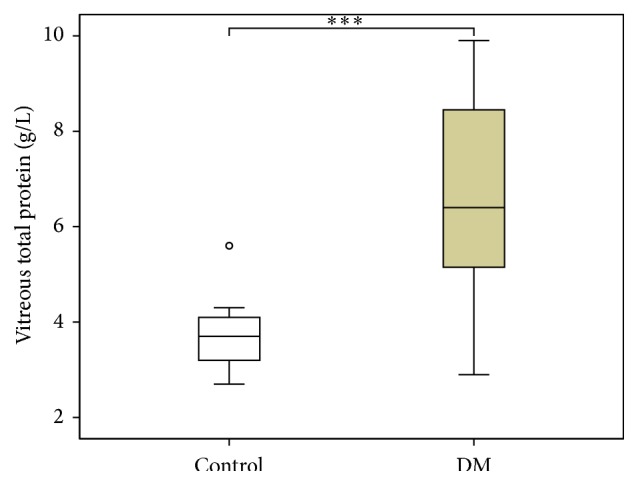
Vitreous concentrations of total protein in diabetic versus control group. DM group *n* = 16, control group *n* = 13, and ^*∗∗∗*^ *p* < 0.001 DM versus control patients.

Biochemical analysis of the vitreous showed significant differences between DM and control group in the concentration of VEGF, UA, and total protein but not albumin as shown in Table 2 and Figures 1 [Fig fig2]–3. In all nondiabetic control subjects, the concentration of VEGF in vitreous was under the detection limit of 31.2 pg/mL.

**Figure 4:**
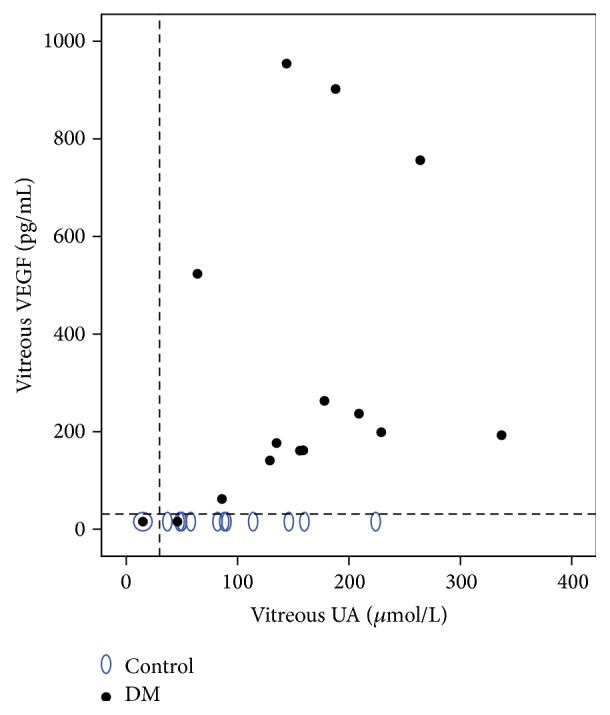
Relationship between vitreous VEGF and vitreous UA concentrations in diabetic versus control group. Dashed lines represent limits of detection (VEGF = 31.2 pg/mL, UA = 30 *µ*mol/L).

In the diabetic group, UA concentration in vitreous correlated significantly with vitreous VEGF concentration (*ƍ* = 0.559, *p* = 0.03). However, in DME vitreous VEGF and UA did not correlate with the total vitreous protein. Further, in the control group, no significant correlation between the biochemical analytes in vitreous was found.  Figure 4 shows the relationship between vitreous VEGF and vitreous UA of DME and control group.

Median of serum concentration of UA in diabetic patients was significantly elevated compared with the control group (337.0 *μ*mol/L, IQR: 324.0–407.0 *μ*mol/L in DM group versus 259.5 *μ*mol/L, IQR: 220.0–334.8 *μ*mol/L in control group; *p* = 0.025). Also median concentration of VEGF in serum of diabetic patients (414.3 pg/mL, IQR: 293.1–512.0 pg/mL) was higher than in controls (332.7 pg/mL, IQR: 149.4–551.8 pg/mL), but the difference was not significant.

There was a significant correlation between UA concentrations in serum and vitreous (*ƍ* = 0.652, *p* = 0.016) in the control group but not in DME. Further, no significant correlation between concentrations of VEGF in serum and vitreous was found in both groups.

#### 3.1.2. OCT Parameters

The median CRT, CAT, and CV did not differ significantly between both groups and are listed in Table 3. Significant difference was found in presence of SRD between the groups as shown in Table 3.

In the diabetic group, there was a significant correlation between CRT and CAT (*ƍ* = 0.589, *p* = 0.016). The CRT of DM subjects also correlated significantly with the CV (*ƍ* = 0.581, *p* = 0.018). However, the strongest correlation in the DM group was between CAT and CV (*ƍ* = 0.999, *p* < 0.001). The SRD was found in the OCT scans of 6 diabetic eyes, but its presence did not correlate with any of the other OCT parameters.

Further, among all OCT parameters, only CV correlated significantly with the concentration of vitreous VEGF in the DM group (*ƍ* = 0.515, *p* = 0.041). The CRT, CAT, CV, and SRD show in DM and control subjects no significant correlation to vitreous concentrations of UA, albumin, or total protein.

The correlation of logMAR BCVA with changes in OCT parameters and vitreous content was also evaluated and we found it to be nonsignificant in both groups. There was also no correlation between OCT parameters and serous concentrations of UA or VEGF.

### 3.2. Discussion

The results demonstrate that biochemical analysis of the vitreous showed significant higher concentrations of VEGF, UA, and total protein in DM and control group. Moreover, in patients with DME intravitreal levels of UA correlate significantly with intravitreal levels of VEGF. Furthermore, we found that the CV measured with Cirrus HD-OCT correlate significantly with the concentration of VEGF in the vitreous of patients with NPDR and DME.

In our earlier study, we showed that the levels of intravitreal UA correlated significantly with the degree of DR [16] and recently also serum UA concentration has been found to be associated with increase in severity of DR [24]. Finding significant higher UA concentration in vitreous of DM compared to controls and a correlation between UA and VEGF in the vitreous of NPDR patients supports our assumption that UA too may be one contributing causal factor in the pathogenesis of DR.

UA is a degradation product of metabolism and under normal conditions UA acts as an antioxidant. In diabetics, hyperglycaemia induces redox stress, which leads to consumption of the naturally occurring local antioxidants protecting capillary endothelium [17]. This results in urate redox shuttle, meaning that UA paradoxically becomes prooxidant and contributes to endothelial dysfunction through oxidative-redox stress [17]. Johnson et al. showed that local ischemia results, via enzymatic activation, in increased UA production as well as oxidant formation [25]. Decreased total antioxidant status was shown to contribute to the progression of PDR via induction of VEGF [26].

On the other side, high UA concentration in the vitreous of diabetic patients may also be a compensatory protective factor. Under experimental conditions, the VEGF-induced production of reactive oxygen species was attenuated by urate; however, it did not modify the VEGF-induced changes in permeability of monolayers [27]. This could explain the correlation of UA and VEGF in the vitreous of diabetic group found in the present study.

It has to be elucidated whether UA is originating from leakage of retinal vessels, which is increased in DR, or from local production. Although total vitreous protein was significantly higher in the diabetic group compared to controls, its level did not correlate with both UA and VEGF. Furthermore, in the diabetic subjects we found no correlation between serum and vitreous level of UA. These findings support the local production of UA in DR. However, to be able to distinguish the origin of increased UA in the vitreous, further analyses, for example, with tagged UA, should be done.

Since there was a correlation between vitreous UA and VEGF but no correlation between vitreous UA and OCT parameters, we conclude that UA has a probable relation with diabetic microangiopathy and accordingly DR but not directly with the development of DME.

Recent studies have shown that VEGF causes conformational changes in the tight junctions of retinal vascular endothelial cells and plays a major role in the elevated vascular permeability in diabetic eyes with DME. It is well known that the vitreous VEGF levels correlate significantly with the severity of DR [11–13, 28]. Few authors have also found a significant correlation between retinal thickness at the fovea measured on OCT and VEGF concentrations in the vitreous [29, 30] and aqueous [31]. The association of vitreous VEGF levels and DME morphology was studied by Sonoda et al. [32] and these authors showed no significant differences in VEGF concentrations in cystoid versus diffuse aspect of DME.

In the present study, there was no significant correlation between vitreous VEGF levels and CRT of diabetic patients. However, the results show that in DME increase in CV correlated with increased concentration of VEGF in the vitreous. Nevertheless, one caveat is that previous studies defined central retinal thickness differently. The retinal thickness at the central fovea in Funatsu et al. [29] was calculated as average foveal thickness from 4 manual measurements per patient. Shimada et al. [30] used the average thickness of the central area with 1000 *μ*m in diameter calculated by Humphrey OCT. Javanmard et al. [31] defined the central macular thickness as the average thickness of the central 500 *μ*m in diameter.

The mean CRT in DME is widely accepted as the new surrogate marker for evaluating treatment efficacy [33]. This is also because the changes in the fovea are deciding for the visual acuity. In our study, the CRT, CAT, and CV in both DM and control group did not correlate with the logMAR BCVA. There are also studies evaluating the effect of the anti-VEGF therapy using both the mean foveal thickness and CV [34]. On the other hand, since DME usually affects the macular area and not only the foveal region, assessment of VEGF concentration in clinical practice using the CV is comprehensible.

Like us, Sonoda et al. showed that there was no significant correlation between intravitreal VEGF levels and the amount of subretinal fluid in DME [35]. Other studies have reported that eyes with serous retinal detachment often have a poor prognosis after treatment [22, 36].

The strength of the study described here is that it determined the relationship between the levels of intravitreal biochemical parameters and retinal morphology at the same time. The limitation was the small sample size (29 eyes). This was caused by decreasing use of vitrectomy for DME and this curtailed collection of vitreous samples. Although our vitrectomy for DME might be considered overtreatment, it was a comparatively effective method as it stabilized the intraocular condition of DME and the efficacy was maintained for a long period [37]. In interpreting or generalizing our results, it should be remembered that the findings demonstrate association of VEGF levels in the vitreous with the cube volume, but they do not prove cause and effect. Further, the study was focused on VEGF and UA; however, the pathogenesis of the DME is complex and still not fully understood.

## 4. Conclusions

Vitreous concentrations of uric acid and VEGF were significantly higher in DM subjects than in controls. Moreover, vitreous UA concentration correlated significantly with the vitreous VEGF concentrations in patients with NPDR and cystoid DME. Increased VEGF concentrations are known to be involved in the pathogenesis of DME. Our results suggest that, apart from VEGF, the role of UA in the pathogenesis and progression of DR should also be considered.

Comparing OCT parameters to the vitreous levels of UA and VEGF, we found that increased concentration of intravitreal VEGF in patients with NPDR and cystoid DME correlated with increase of cube volume calculated by Cirrus HD-OCT. Since DME usually affects the macular area and not only the foveal region, the assessment of the VEGF concentration in clinical practice using the cube volume is comprehensible. This OCT parameter could be used to assess the efficacy of anti-VEGF therapy.

## Figures and Tables

**Table 1:** Clinical and laboratory characteristic of diabetic subjects and nondiabetic controls.

Parameter	DME (*n* = 16)	Control (*n* = 13)	*p*
Number of patients (men/women)	4/12	1/12	ns
Age (years)	71 (61–77)	71 (66–74)	ns
LogMAR BCVA	1.0 (0.6–1.0)	0.5 (0.5–0.6)	*∗∗*
Chronic kidney disease	1 (6.2%)	0 (0.0%)	ns
Dyslipidemia	8 (50.0%)	4 (30.8%)	ns
Hypertension	14 (87.5%)	10 (76.9%)	ns
HbA_1c_ (mmol/mol)	51.5 (43.0–63.3)	NA	NA
Serum albumin (g/L)	42.7 (41.1–44.6)	44.4 (39.6–45.2)	ns
CRP (mg/L)	1.6 (1.0–3.7)	2.0 (0.8–3.6)	ns

Data are expressed as median ± interquartile range or total number and %.

ns: not significant; ^*∗*^ *p* < 0.05, ^*∗∗*^ *p* < 0.01, and ^*∗∗∗*^ *p* < 0.001 DME versus control patients; BCVA: best corrected visual acuity; CRP: C-reactive protein; HbA_1c_: glycated haemoglobin; logMAR: logarithm of the minimal angle of resolution; and NA: not assessed; chronic kidney disease was defined as either structural kidney damage or glomerular filtration rate < 1.0 mL·s^−1^·1.73 m^−2^ for ≥ 3 months.

**Table 2:** Laboratory analysis of vitreous of diabetic subjects and nondiabetic controls.

Parameter	DME (*n* = 16)	Control (*n* = 13)	*p*
VEGF (pg/mL)	192.7 (140.9–523.5)	<LOD	*∗∗∗*
UA (*µ*mol/L)	156.0 (86.0–209.0)	70.0 (48.5–138.0)	*∗*
Albumin (mg/L)	1050 (618–1780)	550 (295–1495)	ns
Total protein (g/L)	6.3 (4.9–9.1)	3.6 (3.1–4.2)	*∗∗∗*

Data are expressed as median with interquartile range.

ns: not significant, ^*∗*^ *p* < 0.05, ^*∗∗*^ *p* < 0.01, and ^*∗∗∗*^ *p* < 0.001 DME versus control patients.

LOD: limit of detection (31.2 pg/mL).

**Table 3:** OCT parameters of diabetic subjects and nondiabetic controls.

Parameter	DME (*n* = 16)	Control (*n* = 13)	*p*
CRT (*µ*m)	479.0 (421.5–661.3)	498.0 (374.5–540.5)	ns
CAT (*µ*mL)	392.0 (329.8–414.3)	332.0 (316.0–346.5)	ns
CV (mm^3^)	14.2 (11.9–15.0)	12.0 (11.4–12.5)	ns
SRD	6 (37.5%)	0 (0%)	*∗*

Data are expressed as median with interquartile range or total number and %.

ns: not significant, ^*∗*^ *p* < 0.05, ^*∗∗*^ *p* < 0.01, and ^*∗∗∗*^ *p* < 0.001 DME versus control patients.

CAT: cube average thickness, CRT: central retinal thickness, CV: cube volume, and SRD: serous retinal detachment.

## References

[1] Sivaprasad S., Gupta B., Crosby-Nwaobi R., Evans J. (2012). Prevalence of diabetic retinopathy in various ethnic groups: a worldwide perspective. *Survey of Ophthalmology*.

[2] Moss S. E., Klein R., Klein B. E. K. (1998). The 14-year incidence of visual loss in a diabetic population. *Ophthalmology*.

[3] Sheetz M. J., King G. L. (2002). Molecular understanding of hyperglycemia's adverse effects for diabetic complications. *The Journal of the American Medical Association*.

[4] Gardner T. W., Antonetti D. A., Barber A. J., LaNoue K. F., Levison S. W. (2002). Diabetic retinopathy: more than meets the eye. *Survey of Ophthalmology*.

[5] Shin E. S., Sorenson C. M., Sheibani N. (2014). Diabetes and retinal vascular dysfunction. *Journal of Ophthalmic and Vision Research*.

[6] Joussen A. M., Poulaki V., Le M. L. (2004). A central role for inflammation in the pathogenesis of diabetic retinopathy. *The FASEB Journal*.

[7] Kowluru R. A., Chan P.-S. (2007). Oxidative stress and diabetic retinopathy. *Experimental Diabetes Research*.

[8] Simó R., Carrasco E., García-Ramírez M., Hernández C. (2006). Angiogenic and antiangiogenic factors in proliferative diabetic retinopathy. *Current Diabetes Reviews*.

[9] Praidou A., Androudi S., Brazitikos P., Karakiulakis G., Papakonstantinou E., Dimitrakos S. (2010). Angiogenic growth factors and their inhibitors in diabetic retinopathy. *Current Diabetes Reviews*.

[10] Tarr J. M., Kaul K., Chopra M., Kohner E. M., Chibber R. (2013). Pathophysiology of diabetic retinopathy. *ISRN Ophthalmology*.

[11] Quam T., Xu Q., Joussen A. M. (2001). VEGF-initiated blood-retinal barrier breakdown in early diabetes. *Investigative Ophthalmology & Visual Science*.

[12] Penn J. S., Madan A., Caldwell R. B., Bartoli M., Caldwell R. W., Hartnett M. E. (2008). Vascular endothelial growth factor in eye disease. *Progress in Retinal and Eye Research*.

[13] Caldwell R. B., Bartoli M., Behzadian M. A. (2003). Vascular endothelial growth factor and diabetic retinopathy: pathophysiological mechanisms and treatment perspectives. *Diabetes/Metabolism Research and Reviews*.

[14] Massin P., Bandello F., Garweg J. G. (2010). Safety and efficacy of ranibizumab in diabetic macular edema (RESOLVE study): a 12-month, randomized, controlled, double-masked, multicenter phase II study. *Diabetes Care*.

[15] Mitchell P., Bandello F., Schmidt-Erfurth U. (2011). The RESTORE Study: ranibizumab monotherapy or combined with laser versus laser monotherapy for diabetic macular edema. *Ophthalmology*.

[16] Krizova L., Kalousova M., Kubena A. (2011). Increased uric acid and glucose concentrations in vitreous and serum of patients with diabetic macular oedema. *Ophthalmic Research*.

[17] Hayden M. R., Tyagi S. C. (2004). Uric acid: a new look at an old risk marker for cardiovascular disease, metabolic syndrome, and type 2 diabetes mellitus: the urate redox shuttle. *Nutrition and Metabolism*.

[18] Massin P., Girach A., Erginay A., Gaudric A. (2006). Optical coherence tomography: a key to the future management of patients with diabetic macular edema. *Acta Ophthalmologica Scandinavica*.

[19] Mushtaq B., Crosby N. J., Dimopoulos A. T. (2014). Effect of initial retinal thickness on outcome of intravitreal bevacizumab therapy for diabetic macular edema. *Clinical Ophthalmology*.

[20] World Health Organization (1999). *Definition, Diagnosis and Classification of Diabetes Mellitus and Its Complications: Report of a WHO Consultation. Part 1: Diagnosis and Classification of Diabetes Mellitus*.

[21] Wilkinson C. P., Ferris F. L., Klein R. E. (2003). Proposed international clinical diabetic retinopathy and diabetic macular edema disease severity scales. *Ophthalmology*.

[22] Deák G. G., Bolz M., Ritter M., Prager S., Benesch T., Schmidt-Erfurth U. (2010). A systematic correlation between morphology and functional alterations in diabetic macular edema. *Investigative Ophthalmology and Visual Science*.

[23] Reznicek L., Cserhati S., Seidensticker F. (2013). Functional and morphological changes in diabetic macular edema over the course of anti-vascular endothelial growth factor treatment. *Acta Ophthalmologica*.

[24] Lee J.-J., Yang I.-H., Kuo H.-K. (2014). Serum uric acid concentration is associated with worsening in severity of diabetic retinopathy among type 2 diabetic patients in Taiwan—a 3-year prospective study. *Diabetes Research and Clinical Practice*.

[25] Johnson R. J., Kang D.-H., Kivlighn S. (2003). Is there a pathogenic role for uric acid in hypertension and cardiovascular and renal disease?. *Hypertension*.

[26] Yokoi M., Yamagishi S.-I., Takeuchi M. (2005). Elevations of AGE and vascular endothelial growth factor with decreased total antioxidant status in the vitreous fluid of diabetic patients with retinopathy. *British Journal of Ophthalmology*.

[27] Marumo T., Noll T., Schini-Kerth V. B. (1999). Significance of nitric oxide and peroxynitrite in permeability changes of the retinal microvascular endothelial cell monolayer induced by vascular endothelial growth factor. *Journal of Vascular Research*.

[28] Zhang X., Zeng H., Bao S., Wang N., Gillies M. C. (2014). Diabetic macular edema: new concepts in patho-physiology and treatment. *Cell & Bioscience*.

[29] Funatsu H., Yamashita H., Nakamura S. (2006). Vitreous levels of pigment epithelium-derived factor and vascular endothelial growth factor are related to diabetic macular edema. *Ophthalmology*.

[30] Shimada H., Akaza E., Yuzawa M., Kawashima M. (2009). Concentration gradient of vascular endothelial growth factor in the vitreous of eyes with diabetic macular edema. *Investigative Ophthalmology and Visual Science*.

[31] Javanmard S. H., Hasanpour Z., Abbaspoor Z., Naderian G. A., Jahanmard M. (2012). Aqueous concentrations of VEGF and soluble VEGF receptor-1 in diabetic retinopathy patients. *Journal of Research in Medical Sciences*.

[32] Sonoda S., Sakamoto T., Yamashita T., Shirasawa M., Otsuka H., Sonoda Y. (2014). Retinal morphologic changes and concentrations of cytokines in eyes with diabetic macular EDEMA. *Retina*.

[33] Diabetic Retinopathy Clinical Research Network (2007). Relationship between optical coherence tomography-measured central thickness and visual acuity in diabetic macular edema. *Ophthalmology*.

[34] Nguyen Q. D., Tatlipinar S., Shah S. M. (2006). Vascular endothelial growth factor is a critical stimulus for diabetic macular edema. *American Journal of Ophthalmology*.

[35] Sonoda S., Sakamoto T., Shirasawa M., Yamashita T., Otsuka H., Terasaki H. (2013). Correlation between reflectivity of subretinal fluid in OCT images and concentration of intravitreal VEGF in eyes with diabetic macular edema. *Investigative Ophthalmology & Visual Science*.

[36] Ota M., Nishijima K., Sakamoto A. (2010). Optical coherence tomographic evaluation of foveal hard exudates in patients with diabetic maculopathy accompanying macular detachment. *Ophthalmology*.

[37] Kumagai K., Furukawa M., Ogino N., Larson E., Iwaki M., Tachi N. (2009). Long-term follow-up of vitrectomy for diffuse nontractional diabetic macular edema. *Retina*.

